# Physiological effect of olfactory stimulation by Hinoki cypress (*Chamaecyparis obtusa*) leaf oil

**DOI:** 10.1186/s40101-015-0082-2

**Published:** 2015-12-22

**Authors:** Harumi Ikei, Chorong Song, Yoshifumi Miyazaki

**Affiliations:** Center for Environment, Health and Field Sciences, Chiba University, 6-2-1 Kashiwa-no-ha, Kashiwa, Chiba 277-0882 Japan; Current Address: Forestry and Forest Products Research Institute, 1 Matsunosato, Tsukuba, Ibaraki 305-8687 Japan

**Keywords:** Japanese cypress, Leaf oil, Odor, Physiological relaxation, Prefrontal activity, Near-infrared spectroscopy, Autonomic nerve activity, Heart rate variability, Semantic differential method

## Abstract

**Background:**

In recent years, increasing attention has been paid to the physiological effects of nature-derived stimulation. The physiological relaxation effects caused by forest-derived olfactory stimuli have been demonstrated. However, there are no studies on the physiological effects of olfactory stimuli by Hinoki cypress (*Chamaecyparis obtusa*) leaves. We investigated the effects of olfactory stimulation by Hinoki cypress leaf oil on the left/right prefrontal cortex activity, assessed using near-infrared time-resolved spectroscopy (TRS), and on the autonomic nervous activity, assessed by measuring heart rate variability (HRV).

**Method:**

Thirteen female university students (mean age, 21.5 ± 1.0 years) participated in the study. Physiological measurements were performed in an artificial climate maintained at 25 °C, 50 % relative humidity, and 230-lx illumination. Hinoki cypress leaf oil was used as an olfactory stimulation with air as the control. The odor was administered for 90 s, while the subjects sat with their eyes closed. Oxyhemoglobin (oxy-Hb) concentrations were measured in the prefrontal cortex using TRS. The high-frequency (HF) component of HRV, which is an estimate of parasympathetic nervous activity, and the low-frequency (LF)/(LF + HF) ratio, which is an estimate of sympathetic nervous activity, were measured by electrocardiography. A modified semantic differential method was used to perform subjective evaluations.

**Results:**

Olfactory stimulation by Hinoki cypress leaf oil induced a significant reduction in oxy-Hb concentration in the right prefrontal cortex and increased parasympathetic nervous activity. The subjects reported feeling more comfortable.

**Conclusion:**

These findings indicate that olfactory stimulation by Hinoki cypress leaf oil induces physiological relaxation.

## Background

In recent years, increasing attention has been paid to the physiological relaxation effects of nature-derived stimulation, and studies on the physiological relaxation effect of forest environments, including viewing forest scenery while sitting, have been performed [1–12]. Sedation of brain activity has been examined by an indicator of total hemoglobin concentration in the prefrontal cortex [1]. Autonomic nervous activity has been investigated by using indicators such as heart rate variability (HRV), heart rate (or pulse rate), and blood pressure. Compared with viewing an urban environment, viewing forest scenery for 15 min can increase parasympathetic nervous activity [2–7], which is enhanced in relaxing situations; suppress sympathetic nervous activity [2–6], which is increased in stress states; reduce blood pressure [3–5, 8]; and decrease heart rate or pulse rate [3–5, 7, 8]. In addition, decreased salivary cortisol concentrations of stress hormones have been reported [1, 3, 4, 6–8]. Similarly, walking in forests can decrease cerebral blood flow in the prefrontal cortex [1], increase parasympathetic nervous activity [3–5, 9], suppress sympathetic nervous activity [3–5, 9], decrease blood pressure [3–5], decrease heart rate or pulse rate [3–5, 9], and decrease salivary cortisol concentration [1, 3, 4] compared with walking in an urban area. In addition, walking in forests or viewing forests has been shown to enhance natural killer cell activity and improve immune function [10]. These effects have been reported to last 1 month [11, 12]. These studies showed that contact with nature caused physiological relaxation effects and improved immune function, which demonstrated the preventive medical effects of forest environments.

Forest environments affect humans through the senses of smell, sight, and hearing. The physiological effects experienced through each sense should be determined. The physiological effects of olfactory stimuli caused by forests or wood have been demonstrated [13–18]. Olfactory stimuli of air-dried wood chips of Hinoki cypress, which is a typical tree of Japan, have been shown to reduce oxyhemoglobin (oxy-Hb) concentrations in the prefrontal cortex [13]. Moreover, Japanese cedar chips have been shown to decrease systolic blood pressure and prefrontal cortex activity [14]. In addition, it has been reported that inhalation of cedrol, which is a compound that occurs in cedar extract, induced parasympathetic nervous activity and reduced sympathetic nervous activity [15]. Inhalation of α-pinene and limonene, which are major components of wood odor, decreased systolic blood pressure [14], and d-limonene enhanced activity of the parasympathetic nervous system and decreased heart rate [16]. A negative correlation between a subjects’ heart rate and their subjective feeling of pleasantness after olfactory stimulation by six essential oil components, including pyridine, l-menthol, and 1,8-cineole, has also been demonstrated [17]. Furthermore, it has been reported that staying for three nights in a Hinoki cypress essential-oil-filled room at an urban hotel induced natural killer cell activity and reduced concentrations of adrenaline and noradrenaline in urine [18]. However, there are no studies on the physiological effects of olfactory stimuli induced by Hinoki cypress leaves.

Hinoki cypress (*Chamaecyparis obtusa*), a coniferous tree, has been used as material in construction and furniture for a long time. The essential oils extracted from leaves and twigs have been used as functional additives or fragrances in soap, toothpaste, and cosmetics. Therefore, the Hinoki cypress and its fragrance are familiar. Previous studies have demonstrated that Hinoki cypress leaf oil or its extraction inhibited the growth of foodborne pathogens [19]; had antibacterial and antifungal effects [20, 21]; exhibited insecticidal activity in stored products against adult insects, such as *Callosobruchus chinensis* (L.) and *Sitophilus oryzae* (L.) [22]; exhibited mosquito larvicidal activity against fourth-stage larvae [23]; and exhibited acaricidal activity against *Dermatophagoides* spp. [24, 25]. However, to the best of our knowledge, no study has evaluated the physiological effects of Hinoki cypress leaf oil odor on the prefrontal cortex and on the autonomic nervous activity.

In this study, we investigated the effects of olfactory stimulation by the Hinoki cypress leaf oil on the left and right prefrontal cortex activity, which was assessed using near-infrared time-resolved spectroscopy (TRS), and on the autonomic nervous activity assessed by HRV.

## Methods

### Participants

The study participants were 13 female university students (mean age, 21.5 ± 1.0 years). Subjects who were being treated for disease or who were menstruating during the study period were excluded. All subjects were informed about the aim and procedures involved in the experiment, and they provided written informed consent to participate. This study was performed in accordance with the regulations of the Ethics Committee of the Center for Environment, Health and Field Sciences, Chiba University, Japan (project identification code number: 5).

### Study protocol

**Fig. 1:**
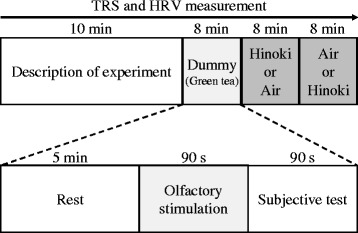
Experimental protocol

Physiological measurements were performed in a chamber with an artificial climate maintained at 25 °C, 50 % relative humidity, and 230-lx illumination. In the waiting room, the subjects received a description of the experiment, signed the agreement, and then moved into the climate-controlled chamber. After fitting the sensors for physiological measurement, subjects received a description of the measurement procedure again for 10 min while sitting. Next, the subjects received a practice dummy stimulation (smell of green tea leaves). The subjects then rested by sitting with their eyes closed for 30–60 s; thereafter, the experimental odor was administered for 90 s. The subjective evaluation test was performed after odor administration using the same parameters as in the practice dummy stimulation. Figure 1 shows the experimental protocol. A within-subject experiment to eliminate the effect of the order of olfactory stimulation was performed. Seven participants were administrated stimuli in the following order: Hinoki cypress leaf oil and then control (air). The remaining six participants received control (air) first and then Hinoki cypress leaf oil.

### Olfactory stimulation

**Fig. 2:**
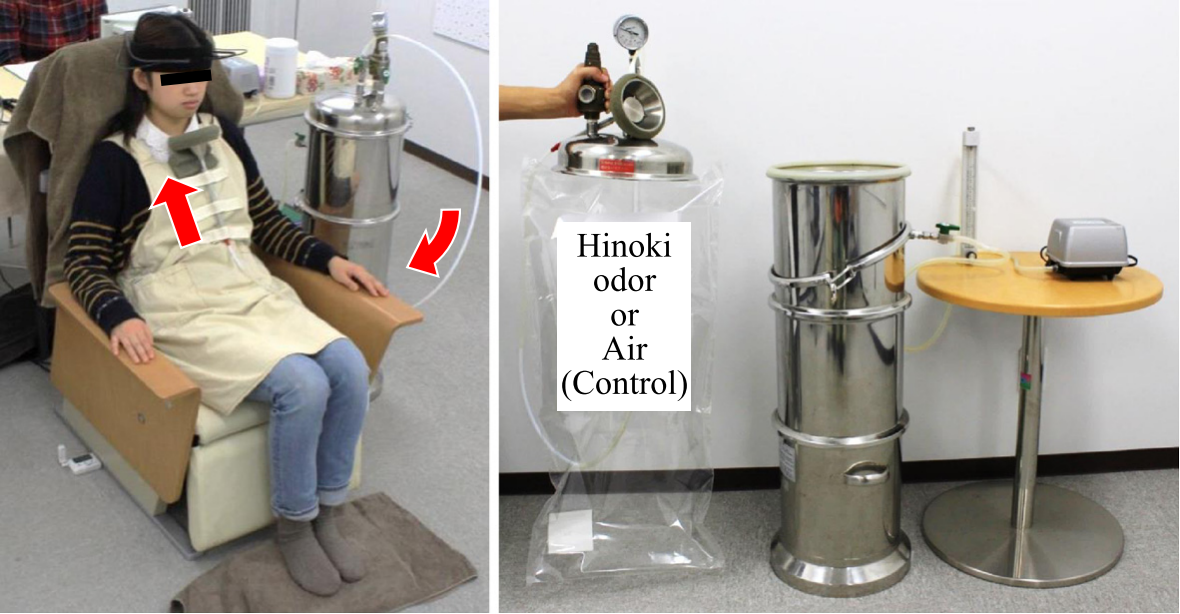
Olfactory stimulation procedure and device used to administer the odors

Hinoki cypress leaf oil (*C. obtusa*; Kiseitec Co., Wakayama, Japan) derived from leaves and twigs of Hinoki cypress trees growing in Wakayama Prefecture, Japan, was extracted by steam distillation and used as the olfactory stimulant; air was used as the control. Hinoki cypress leaf oil (2 μL) injected into a 24-L odor bag (polyethylene terephthalate film heat seal bag; NS-KOKEN Co., Ltd., Kyoto, Japan). The odor bag was exposed approximately for 1 h at room temperature to diffuse the essential oil into the bag. Odors were presented to each participant by means of a device fixed on the chest and situated approximately 10 cm under the nose (Fig. 2). The flow rate of the air saturated with the essential oil was set at 3 L/min. Preliminary investigations determined that the subjective intensity of the odor was sensed as “weak” or “easily” sensed.

### Physiological measurement

#### Near-infrared time-resolved spectroscopy

As an indicator of brain activity, TRS, which is a near-infrared spectroscopy method, was used. The sensors were mounted on the subject’s forehead, and oxyhemoglobin (oxy-Hb) concentrations in the prefrontal cortex were measured (TRS-20 system; Hamamatsu Photonics K.K., Shizuoka, Japan) [26–28]. The oxy-Hb concentrations in the left and right prefrontal cortex were measured at 1 Hz for 10 s before (premeasurement condition) odor administration and during the 90 s of odor administration (postmeasurement condition). The data were transformed by linear interpolation because much of the data was measured at approximately 1.0–1.2 s. In addition, each datum was calculated as the difference between the averages of the previous 10-s values.

#### Heart rate variability

As an indicator of autonomic nervous activity, HRV was analyzed for the periods between consecutive R waves (R-R intervals) on electrocardiograms measured by a portable electrocardiograph (Activtracer AC-301A; GMS, Tokyo, Japan) [29, 30]. The power levels of the low-frequency (LF: 0.04–0.15 Hz) and high-frequency (HF: 0.15–0.40 Hz) components of HRV were calculated using the maximum-entropy method (MemCalc/Win; GMS, Tokyo, Japan). The HF power reflected parasympathetic nervous activity. The LF/(LF + HF) ratio reflected sympathetic nervous activity. The means of the data were acquired for 30 s before odor administration and during the 90 s of odor administration.

### Psychological measurement

To subjectively evaluate the psychological effect of the odor, the participants were tested by the modified semantic differential (SD) method [31]. Three pairs of adjectives were assessed on 13 scales as “comfortable–uncomfortable,” “natural–artificial,” and “relaxed–awakening.”

### Statistical analysis

One participant in the TRS and two participants in the HRV were excluded because of data collection errors. All statistical analyses were performed using Statistical Package for Social Sciences software version 20.0 (IBM Corp., Armonk, NY, USA). A paired *t* test was used to compare the physiological responses to the Hinoki cypress leaf oil and control. The Wilcoxon signed-rank test was applied to analyze differences in psychological indices between the responses to the Hinoki cypress leaf oil and control. In all cases, the significance level was set at *P* < 0.05.

In previous reports, the physiological and psychological relaxation effects of nature-derived stimulation have been shown [1–9, 13, 14, 16, 31–35]. One-sided tests were used in this study because we hypothesized that humans would be relaxed by the olfactory stimulation by Hinoki cypress leaf oil.

## Results

### Physiological effects

#### Near-infrared time-resolved spectroscopy

**Fig. 3:**
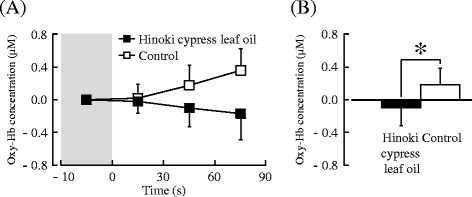
The 30-s averages and overall mean oxy-Hb concentrations in the right prefrontal cortex during olfactory stimulation by Hinoki cypress leaf oil or control. **a** Changes in each 30-s average oxy-Hb concentration over 90 s. **b** Overall mean oxy-Hb concentration. Data are expressed as the mean ± standard error, *n* = 12, *P < 0.05 as determined by the paired *t* test (one-sided)

The changes in the oxy-Hb concentration in the right prefrontal cortex during olfactory stimulation by the Hinoki cypress leaf oil or control are shown in Fig. 3a. A comparison of the mean oxy-Hb concentrations in the right prefrontal cortex after 90 s of olfactory stimulation between the Hinoki cypress leaf oil and control is shown in Fig. 3b. The mean oxy-Hb concentration in the right prefrontal cortex was −0.10 μM after exposure to the Hinoki cypress leaf oil and 0.18 μM after exposure to the control. Olfactory stimulation by the Hinoki cypress leaf oil significantly reduced the oxy-Hb concentration in the right prefrontal cortex relative to that of the control (Fig. 3b, *P =* 0.043).

**Fig. 4:**
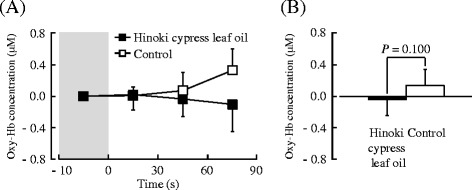
The 30-s averages and overall mean oxy-Hb concentrations in the left prefrontal cortex during olfactory stimulation by Hinoki cypress leaf oil or control. **a** Changes in each 30-s average oxy-Hb concentration over 90 s. **b** Overall mean oxy-Hb concentration. Data are expressed as the mean ± standard error, *n* = 12, determined by the paired *t* test (one-sided)

Figure 4 shows the results in the left prefrontal cortex during olfactory stimulation by the Hinoki cypress leaf oil or control. The results were similar to those in the right prefrontal activity. A comparison of the mean oxy-Hb concentrations in the left prefrontal cortex between 90 s of olfactory stimulation by the Hinoki cypress leaf oil and by the control is shown in Fig. 4b. The difference was not significant, but the results suggested that the Hinoki cypress leaf oil induced a trend toward reduced oxy-Hb concentration in the left prefrontal cortex (Hinoki cypress leaf oil, −0.04 μM; control, 0.14 μM, Fig. 4b; *P* = 0.100).

#### Heart rate variability

**Fig. 5:**
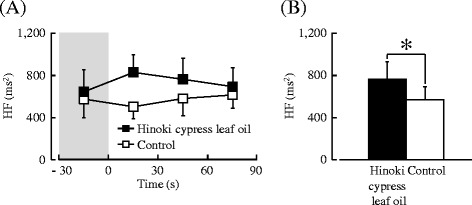
The 30-s averages and overall mean high-frequency (HF) component of the heart rate variability (HRV) during olfactory stimulation by Hinoki cypress leaf oil or control. **a** Changes in each 30-s average HF value over 90 s. **b** Overall mean HF values. Data are expressed as the mean ± standard error, *n* = 11, *P < 0.05 as determined by the paired *t* test (one-sided)

The HF value associated with olfactory stimulation by the Hinoki cypress leaf oil is shown in Fig. 5a. The mean baseline HF did not differ significantly between the Hinoki cypress leaf oil (645.5 ms^2^) and control (575.9 ms^2^) for 30 s before stimulation (premeasurement condition) (*P* = 0.133).

Figure 5b shows the overall mean of the HF value associated with olfactory stimulation by the Hinoki cypress leaf oil. When the results of the HRV power level data were compared, a significant difference was found in the HF power level between the Hinoki cypress leaf oil and control (*P* = 0.020). The HF power level of Hinoki cypress leaf oil (762.1 ms^2^) was 34.5 % higher than that of the control (566.8 ms^2^). It was clear that olfactory stimulation by the Hinoki cypress leaf oil induced a significant increase in parasympathetic nervous activity and thereby induced physiological relaxation.

However, no significant difference was found in the LF/(LF + HF) ratio between the two stimuli (Hinoki cypress leaf oil, 0.41; control, 0.43) (*P* = 0.426).

### Psychological effects

**Fig. 6:**
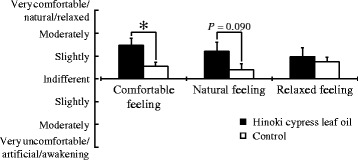
Subjective feelings measured by the modified semantic differential method after olfactory stimulation by Hinoki cypress leaf oil or control. Data are expressed as the mean ± standard error, *n* = 13, *P < 0.05 as determined by the Wilcoxon signed-rank test (one-sided)

The modified SD method was used to provide subjective reports of “comfortable,” “natural,” and “relaxed” feelings (Fig. 6). The subjects provided subjective reports of feeling “slightly comfortable” and “moderately comfortable” for the Hinoki cypress leaf oil; however, they provided reports of “slightly comfortable” and “indifferent” for the control. The response to the Hinoki cypress leaf oil was, therefore, perceived as being significantly more comfortable than that to the control (Fig. 6 left, *P* = 0.009). The difference was not significant, but the results suggested that the Hinoki cypress leaf oil was more natural than the control (Fig. 6 central, *P* = 0.090). However, there was no significant difference in the relaxed feeling between the two stimuli (Fig. 6, right, *P* = 0.251).

## Discussion

This study aimed to clarify the effects of olfactory stimulation by the Hinoki cypress leaf oil on the left and right prefrontal cortex activity, which was assessed by measuring oxy-Hb by TRS, and on the autonomic nervous activity, which was assessed by measuring HRV. These results showed that olfactory stimulation with Hinoki cypress leaf oil significantly decreased oxy-Hb concentration in the right prefrontal cortex and significantly increased parasympathetic nervous activity according to the 90-s overall mean values.

Concerning brain activity, it has been reported that olfactory stimulation by air-dried wood chips of Hinoki cypress reduced oxy-Hb concentrations in the prefrontal cortex [13] and that Japanese cedar chips reduced total Hb concentrations in the prefrontal cortex [14]. In addition, concerning autonomic nervous activity, it has been reported that the inhalation of d-limonene, which is one of the main components of Hinoki cypress leaf oil [36], enhanced parasympathetic nervous activity and decreased heart rate [16]. Inhalation of cedrol, which is an extraction component of cedar oil, induced parasympathetic nervous activity and reduced sympathetic nervous activity [15]. Our findings are consistent with those of previous studies [13–16].

Regarding the subjective evaluations, the subjects felt more comfortable after olfactory stimulation by the Hinoki cypress leaf oil than by the control. However, no significant difference was found in the relaxed feeling. Our previous study reported that olfactory stimulation by rose and orange oil was perceived as being significantly more comfortable and relaxing than that by the control [32]. However, there was no significant difference in the subjective relaxed feeling in this study. Although the reason is unclear, it is known that sabinene, which is one of the main components of Hinoki cypress leaf oil [20], is a pungent component of black pepper [37], so there is a possibility that sabinene affected the subjective evaluations. In addition, we found that there was a tendency toward an increased feeling of being relaxed when the subjective perceived intensity of the odor decreased (*r* = −0.523, *P* = 0.066). Therefore, if the concentration of the Hinoki cypress leaf oil was lower, the subjects might feel more relaxed after olfactory stimulation by the Hinoki cypress leaf oil.

Previous studies have revealed that exposure to a forest environment induced physiological relaxation [1–9] and improved the immune function [10–12]. However, regular contact with natural environments is difficult in many modern societies. Tree-derived essential oils can easily be incorporated into daily life. In recent years, aromatherapy has become popular and is recognized as a general relaxation method [38]. The accumulation of data on the physiological effects of nature-derived olfactory stimuli, such as wood [13–18], flowers [32, 33], plants [34], and fruit [32, 35], has been promoted from the viewpoint of evidence-based medicine. In this study, we clarified the physiological relaxation effects of olfactory stimulation by Hinoki cypress leaf oil. These results may contribute to the improvement in the quality of life of modern people by accumulating scientific evidence on the physiological effects of nature-derived odors, such as those of the Hinoki cypress.

This study had three limitations. First, although this study evaluated brain activity and autonomic nervous activity, other experimental indices, such as natural killer cell activity and stress hormone levels, should be assessed to more comprehensively evaluate the physiological effects of olfactory stimulation by Hinoki cypress leaf oil. Second, the subjects of this study were all female university students aged in their 20s. Studies on other types of subjects, such as males, children, and the elderly, are therefore required. Third, this study only measured the physiological effects resulting from the presentation of olfactory stimuli over a short time period of 90 s. It would, therefore, be useful to study the physiological response due to long-term olfactory stimulation in the future by measuring physiological effects after longer periods of time.

## Conclusions

Olfactory stimulation by Hinoki cypress leaf oil significantly decreased oxy-Hb concentration in the right prefrontal cortex, which is associated with prefrontal cortex activity, and significantly increased the HF component of HRV, which is associated with parasympathetic nervous activity. These findings indicate that olfactory stimulation by Hinoki cypress leaf oil induces physiological relaxation.

## Abbreviations

HF: high frequency
HRV: heart rate variability
LF: low frequency
Oxy-Hb: oxyhemoglobin
SD: semantic differential
TRS: near-infrared time-resolved spectroscopy
